# DEVELOPMENT AND VALIDATION OF THE COUPLE EFFICACY TO BALANCE WORK AND ELDERCARE SCALE

**DOI:** 10.1093/geroni/igad104.3271

**Published:** 2023-12-21

**Authors:** Eunae Cho, Tammy Allen, Grand Cheng

**Affiliations:** National Chengchi University, Taipei City, Taipei, Taiwan (Republic of China); University of South Florida, Tampa, Florida, United States; National University of Singapore, Singapore, Not Applicable, Singapore

## Abstract

This study developed and validated a measure of couple efficacy to balance work and eldercare (i.e., a couple’s shared belief in their conjoint abilities to achieve work-eldercare balance as a couple), a key cognitive factor for work-eldercare arrangements, individual well-being, and work and couple outcomes. Survey data were collected from 408 dual-earner couples in Taiwan who care for an older adult. Exploratory factor analysis identified three factors with eigenvalues greater than 1, but parallel analysis suggested extracting two factors. After considering item contents and factor loadings, a final 2-factor scale comprising 10 items was selected. The first dimension, task-oriented, reflects couple efficacy concerning the execution of various tasks to balance paid work and eldercare (e.g., “I am confident that my spouse/partner and I can work together as a team to devote enough attention to important job and eldercare activities”; α=.81). The second dimension, relationship-oriented, reflects couple efficacy concerning the maintenance of intimate relationship while balancing paid work and eldercare (“I am confident that my spouse/partner and I can work together as a team to support each other in times of stress in trying to balance paid work and eldercare”; α=.87). Confirmatory factor analysis indicated that a hypothesized 2-factor outperforms an alternative 1-factor model. The nomological network involving generalized self-efficacy and life/job/marital satisfaction supported the validity of the new scale. This study contributes to the literature by introducing a new construct and offering a psychometrically sound measure that propels more empirical research on the important population of working informal caregivers.

